# Human-in-the-Loop Suicide Risk Detection and Clinical Escalation in a Generative AI Mental Health Chatbot: Prospective Implementation Study

**DOI:** 10.2196/106118

**Published:** 2026-10-23

**Authors:** Suzette Glasner, Darcy Michero, Matthew Stewart

**Affiliations:** 1 Department of Clinical Affairs Digital Therapeutics, Inc New York, NY United States; 2 Department of Psychiatry David Geffen School of Medicine University of California, Los Angeles Los Angeles, CA United States

**Keywords:** mental health chatbot, digital health, AI, suicidal ideation, suicide screening, human-in-the-loop

## Abstract

**Background:**

Mental illness is a leading cause of disability worldwide, affecting more than 1 billion people, and suicide accounts for 720,000-800,000 deaths annually. Although AI-native mental health chatbots have the potential to help address the substantial gap between treatment needs and workforce capacity, their rapid proliferation has outpaced the development of scalable procedures for identifying and responding to suicidal ideation. Although recommendations call for human oversight to support safety monitoring and intervention in AI-based care, research on human clinical oversight and crisis-response procedures remains limited.

**Objective:**

This study aims to implement a human-in-the-loop (HITL) protocol for suicide risk screening, detection, and clinical escalation in a generative AI (GenAI) mental health chatbot.

**Methods:**

In this implementation study of a HITL suicide risk detection and escalation protocol, adults were recruited online to participate in a single-arm trial of an AI-native mental health chatbot. Participants completed baseline screening using the 9-item Patient Health Questionnaire (PHQ-9). In phase 1, participants who endorsed suicidal ideation were referred to a human clinician for further assessment, safety planning, and provision of referral resources, as appropriate. In phase 2, escalation was triggered by either PHQ-9 responses or the detection of spontaneous, implicit, or explicit disclosures of suicidality during chatbot interactions. In both phases, the Ask Suicide-Screening Questions guided clinician-led assessment and risk stratification. Outcomes included the prevalence of suicidal ideation, the rate of successful clinician outreach, the time from risk detection to initiation of clinician outreach, and the results of risk stratification.

**Results:**

In phase 1, 624 participants completed suicide risk screening, of whom 81 (13%) endorsed PHQ-9 item 9 (score ≥1). Clinician outreach was initiated, on average, 11.2 minutes (SD 13.2 minutes; range 1-54 minutes) after the alert, and 25 of the 81 (31%) participants were successfully reached. In phase 2, 650 participants completed screening, of whom 133 (20.5%) screened positive at baseline. During the 30-day postbaseline observation period, 284 risk-alert events were generated among 172 (26.5%) participants, including 50 conversational suicidal ideation alerts. Clinicians successfully reached 73 of the 172 (42.4%) participants. The mean time from risk detection to initiation of clinician outreach was 11.5 minutes (SD 11.7 minutes; range 1-58 minutes).

**Conclusions:**

Proactive, universal suicide risk screening and conversational detection using a HITL protocol can be operationally implemented in a GenAI mental health chatbot. A subset of participants who were successfully reached by a human clinician described the approach as acceptable and helpful. Given that clinical contact and assessment were not completed for a substantial proportion of alerted participants, further research is needed to optimize assessment and follow-up strategies for individuals who cannot be reached and to characterize the impact of these procedures on clinical outcomes. Nevertheless, the safety procedures described herein provide a framework for developing and refining HITL safety responses and protocols for purpose-built, AI-native mental health chatbots.

## Introduction

Mental health conditions are the leading cause of disability worldwide, affecting 1.17 billion individuals [1], and suicide remains a major contributor to premature mortality, accounting for more than 720,000 deaths annually. With the prevalence of mental illness nearly doubling over the past several decades, there is a growing mismatch between the demand for mental health services and the capacity of the behavioral health workforce [1]. As the need for innovative, scalable intervention approaches to address this shortfall has become increasingly apparent, digital health solutions have rapidly proliferated in behavioral health. Most recently, generative AI (GenAI) applications in mental health have become a focus of growing interest [2], particularly given data indicating substantial use of GenAI for mental health purposes, with nearly half of users with mental health conditions turning to these tools for psychological support [3]. Moreover, a handful of studies suggest that GenAI tools targeting mental health can improve psychological symptoms and well-being [4,5].

Despite the potential advantages of GenAI as a mental health care modality, including improved treatment accessibility and personalized care, the risks of GenAI applications in mental health care are receiving increasing research attention, particularly regarding the detection and management of suicidal ideation. As a novel mental health intervention modality, GenAI crisis response lacks established best practice standards, although some state-level regulatory frameworks are emerging (eg, in California [6]). Moreover, although few studies have evaluated the responses of GenAI mental health applications to suicidality, the largest investigation to date evaluated 29 chatbots, including apps that directly targeted mental health, and found that none met the criteria for acceptable detection and management of suicidal ideation [7]. In light of these findings, along with research indicating that mental health chatbots are slow to escalate suicidality risk scenarios, potentially compromising patient safety [8], clinical oversight and human escalation have been identified as critical considerations in mitigating safety risks associated with GenAI mental health chatbots [9,10].

Human-in-the-loop (HITL) risk detection and escalation models have been recommended as an important safety measure in AI-assisted mental health care [9-13]. In practice, HITL involves engaging licensed clinicians to interpret and intervene when an AI tool encounters a clinical care need or crisis that falls outside its scope of competence to evaluate or manage. Although studies indicate strong consensus among both clinicians [12] and GenAI users [13] regarding the importance, desirability, and appropriateness of HITL models, relatively few GenAI mental health chatbots employ these models, and published studies detailing procedures for human clinical oversight are lacking. In addition, the prevalence of suicidality among individuals who use GenAI mental health chatbots remains largely unknown, leaving the clinical capacity required to support HITL models poorly understood. To address this gap, this 2-phase study employed a digitally administered suicidality screening procedure in the first phase and a conversational suicidality risk detection mechanism in the second phase, coupled with a HITL clinical escalation and response protocol to provide crisis support and mitigate safety risks among adults receiving care from a GenAI mental health chatbot. The study objectives were to (1) characterize the frequency of suicidality, as assessed by universal screening and conversational risk alerts, among adults who use a GenAI mental health chatbot; (2) describe the feasibility (ie, screening completion, alert delivery, time to outreach, contact success rates, and further clinical assessment) and acceptability, based on qualitative data gathered from participants who screened positive, of implementing both a universal suicidality screening measure and a detection system with human clinical oversight to support escalation; and (3) describe the emergency care needs of the target population based on suicidality risk stratification.

## Methods

### Study Design and Participants

This study was conducted in 2 phases with 2 separate cohorts as part of a larger open-label feasibility trial evaluating a GenAI mental health chatbot (Sona; Multimedia Appendices 1 and 2). The chatbot was designed to support psychological well-being and related holistic wellness goals through personalized conversations delivered via voice or text. Participants were recruited through online platforms that provide access to a diverse pool of potential research participants (Prolific [Prolific Academic Ltd] and CloudResearch [CloudResearch LLC]). In phase 1, adults aged 18 years or older were eligible to participate if they were currently employed or had health insurance, spoke English, resided in the United States, had access to a smartphone capable of downloading the study app, and had not participated in a previous Sona study. In phase 2, because suicidal thoughts may occur relatively infrequently in a wellness population, an additional eligibility criterion was introduced to ensure that the sampling approach would yield a sufficient number of conversational disclosures to Sona for evaluating the detection pathway: a symptom severity score greater than 5 (mild or higher severity) on either the 9-item Patient Health Questionnaire (PHQ-9) or 7-item Generalized Anxiety Disorder Scale (GAD-7). See Multimedia Appendix 3 for the iCHECK-DH (I Checked – Digital Health) checklist completed for this study.

During phase 1 recruitment in April 2026, 1388 individuals completed the eligibility prescreening, 784 consented to participate and downloaded the app, and 624 completed the baseline measures. During phase 2 recruitment in July 2026, 1000 individuals completed the eligibility prescreening, 672 consented to participate and downloaded the app, and 650 completed the baseline measures.

As this was a single-arm implementation study, feasibility was evaluated descriptively using the following operationally defined indicators: (1) suicide risk screening completion, defined as the proportion of consented participants who completed baseline screening; (2) alert delivery, defined as the proportion of positive screens that generated an alert to the crisis intervention team, reported separately by detection pathway (ie, PHQ-9 item 9 vs conversational detection); (3) protocol fidelity, defined as adherence to the outreach protocol; (4) staffing, defined by the clinical team composition and corresponding hours of operation; (5) unsuccessful outreach, defined as the proportion of positive screens for which participants could not be reached through human outreach; and (6) technical failures, defined as any instance of alert delivery failure or system downtime that affected the escalation pathway. To describe acceptability, a subgroup of phase 2 participants who triggered a safety alert (4 PHQ-9 alerts and 3 CRISIS_988 alerts) were invited to participate in an optional 60-minute qualitative interview. Participants had consented to these interviews at study enrollment, and each received a US $50 gift card as compensation. The interviews explored participants’ experiences with the safety-alert process, including their experiences answering safety questions while using Sona and being contacted by a clinician for safety and suicide risk assessment and safety planning, if indicated. The interviews also explored whether clinician outreach influenced participants’ attitudes toward or use of the app, their preferences regarding the method of contact, and whether the outreach was expected or came as a surprise.

Given the descriptive nature of the feasibility and acceptability evaluation, no formal power calculation was performed. The target sample size was chosen to provide a stable estimate of the prevalence of suicidal ideation and to yield a sufficient number of clinical escalations to evaluate the feasibility of implementing the suicide risk detection and escalation protocol. Drawing on our prior suicidality screening study, in which a screen-positive rate of approximately 8% was observed [14], we set a target of 500 participants completing baseline screening in each phase, a sample size expected to generate approximately 40 escalation events per phase. As remote, online digital health studies are characterized by substantial attrition, ranging from 30% to 70% [15,16], the recruitment pool was deliberately targeted to be 50% larger than the number of participants needed to implement the safety protocol among at least 8% of participants.

### Ethical Considerations

Before the baseline assessment, informed consent was obtained digitally for participation in the parent pilot feasibility study of the Sona chatbot, a benign wellness tool designed to support psychological well-being and holistic health-related goals. The parent study was granted exempt status by the WCG Institutional Review Board under 45 CFR 46.104(d)(3) on December 14, 2025 (institutional review board approval number 20254520). The safety assessment and escalation procedures reported herein are components of Sona’s standing clinical safety protocol, which is based on well-established standards of care for suicide risk assessment and crisis response across a range of treatments and services, including benign wellness interventions (eg, Joint Commission National Patient Safety Goal 15.01.01: screening, brief severity assessment, safety planning, and escalation). These procedures constitute standard clinical care and were neither introduced nor modified or withheld for research purposes. The parent study was a nonrandomized, single-arm feasibility study, and this report addresses only formative objectives: evaluating the feasibility and acceptability of implementing Sona’s safety protocol. No efficacy hypotheses were tested. Accordingly, the study was not prospectively registered as a clinical trial.

All data were stored on secure, password-protected servers. No personally identifiable information is reported in this study, and data are presented in aggregate to safeguard participants’ privacy and prevent the identification of individual participants.

### Participant Procedures

After downloading the Sona app and providing informed consent, all participants completed digital assessments within the app and via survey. These assessments included questionnaires covering demographics, mental health symptom severity (ie, depressive and anxiety symptoms), and suicidal thoughts and behaviors. Depressive and anxiety symptoms were assessed at baseline and at the 30-day follow-up using the PHQ-9 [17] and the GAD-7 [18]. Health-related quality of life was measured using the 4-item Centers for Disease Control and Prevention Health-Related Quality of Life (CDC HRQOL-4) [19], a measure assessing self-rated general health that yields 3 component scores: physically unhealthy days, mentally unhealthy days, and activity limitation days, each reported as the number of days in the past 30 days. A summary “unhealthy days” score is calculated by combining the physically and mentally unhealthy days scores, with a maximum of 30 days.

### Suicidality Measurement, Risk Detection, and Escalation

#### Overview

Before suicidality screening began, participants were informed within the app that if they reported symptoms raising safety concerns, a member of the clinical care team would contact them by telephone or attempt to reach them through other means to provide support. The clinical team responsible for responding to all escalations included counselors trained in administering the Ask Suicide-Screening Questions (ASQ), interpreting results, and responding to both acute positive screens (ie, current suicidal ideation) and nonacute positive screens (ie, no current suicidal ideation).

Screening for suicidal ideation and risk was first conducted using item 9 of the PHQ-9, which asks respondents to rate how often they have been bothered in the past 2 weeks by “thoughts that you would be better off dead or of hurting yourself in some way,” on a scale from 0 (not at all) to 3 (nearly every day). The PHQ-9 was presented as a fixed digital questionnaire; therefore, the GenAI chatbot did not select, generate, reword, interpret, or score its items. In phase 1, the PHQ-9 was administered during business hours (8 AM-7:30 PM ET, Monday-Friday) to ensure that positive screens could be escalated to a human clinician in real time. In phase 2, human clinical coverage was expanded to 24 hours per day, 5 days per week, with PHQ-9 administration occurring during these hours. Any item 9 score of 1 or greater triggered Sona’s digital suicidality risk detection and escalation process. This process was supported by a system embedded in the Sona platform. Participants who screened positive for suicidality immediately received a pop-up message informing them that a member of the Sona team would contact them to provide support. The message also provided 24/7 crisis and suicidality intervention resources (ie, Lifeline or 988) that participants could call or SMS text message at any time if they needed or wanted support. Concurrently, the system sent a notification to a clinical safety team channel on Sona’s instant messaging platform (Slack; Slack Technologies) to prompt participant outreach (Figure 1). This escalation system operates as a proprietary, closed-loop notification pathway rather than a standards-based interoperability framework and does not currently use interoperability standards such as HL7 FHIR (Health Level Seven International Fast Healthcare Interoperability Resources) for data exchange with external electronic health record systems.

**Figure 1:**
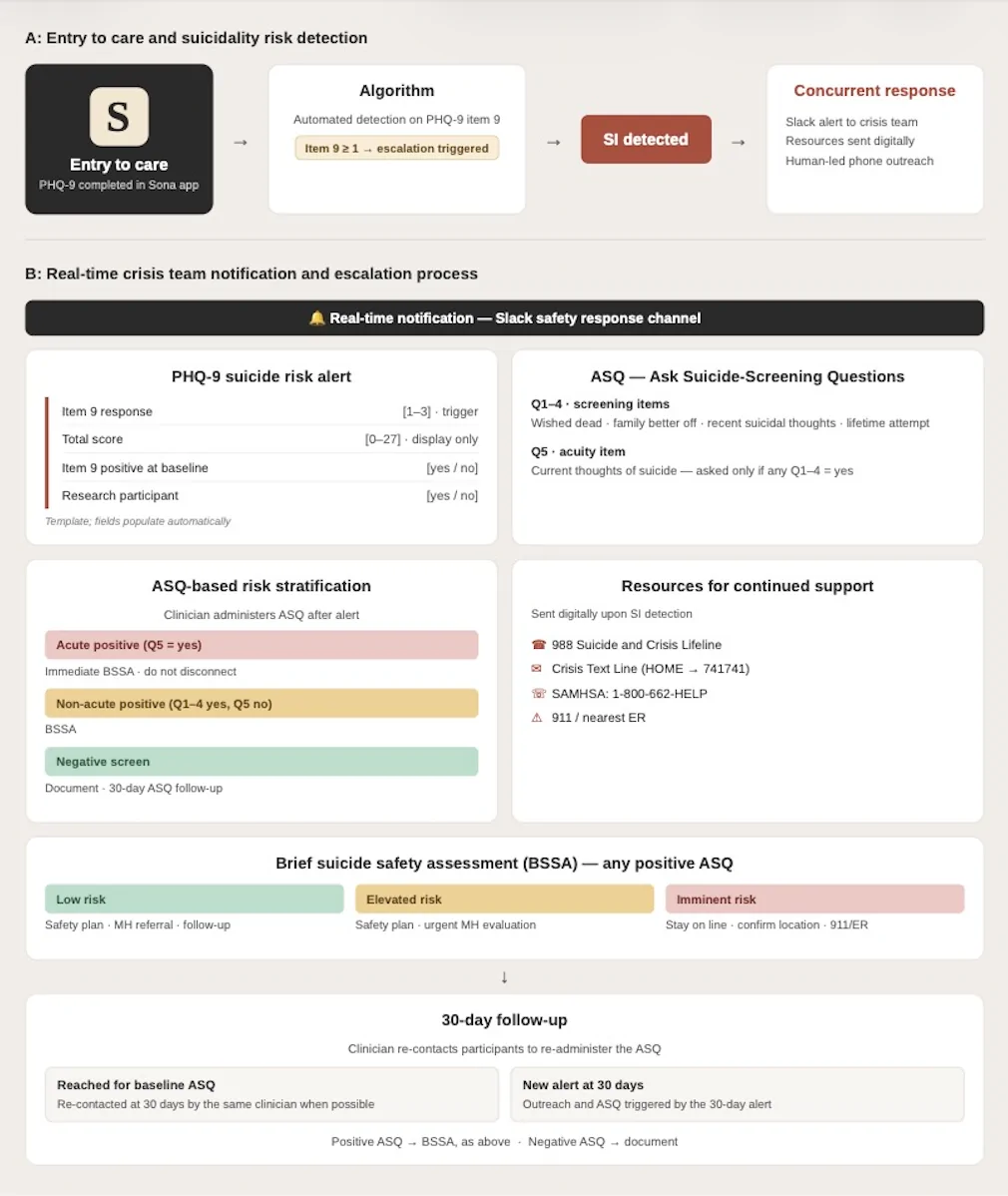
Workflow of the Sona digital suicidality detection and crisis response protocol. (A) A response of 1 or higher to item 9 of the 9-item Patient Health Questionnaire (PHQ-9) triggers a suicidal ideation (SI) alert, concurrent notification of the crisis team, delivery of resources, and telephone outreach. (B) A clinician administers the Ask Suicide-Screening Questions (ASQ); a positive screen prompts a Brief Suicide Safety Assessment (BSSA). At 30 days, the ASQ is readministered to participants reached at baseline and to those who trigger a new positive alert based on PHQ-9 item 9. MH: mental health; NIMH: National Institute of Mental Health.

Upon notification, the first clinician in the crisis intervention group to initiate outreach to a participant who screened positive for suicidality posted a digital indicator (ie, an “eyes” emoji) to inform the other group members that clinician assignment and intervention had begun. The self-designated clinician contacted participants who screened positive on PHQ-9 item 9 by telephone to conduct further assessment using the Ask Suicide-Screening Questions (ASQ), a 4-item suicide risk screening tool designed to detect recent suicidal ideation and a lifetime history of suicide attempts [20] (Figure 1). The measure has been validated among medical patients [20] and successfully implemented in virtual behavioral health care settings [14]. A positive screen is defined as endorsement of any of the 4 items, whereas a response of “no” to all 4 items constitutes a negative screen. Respondents who screen positive on any of the first 4 items are asked a fifth question (“Are you having thoughts of killing yourself right now?”) to assess acute suicidal ideation. A positive response to the fifth item indicates an “acute positive” screen, whereas a positive response to any of the first 4 items and a negative response to the fifth item indicate a “nonacute positive” screen. All screens were scored in real time, and positive screens were immediately followed by the ASQ Brief Suicide Safety Assessment (BSSA) to further triage suicide risk and develop an individualized safety plan for review with the participant, as indicated [21]. Mental health referrals were provided as appropriate, based on a list of state-specific crisis and mental health resources that had been compiled and shared electronically with staff before study implementation. If a participant could not be reached by telephone, crisis and mental health resources were sent by email, and up to 3 additional telephone outreach attempts were made over the next 3 days. All participants received a digital follow-up PHQ-9 assessment 30 days after entry into care. Outreach was reinitiated for repeat ASQ screening and intervention, as appropriate, for participants with a new positive PHQ-9 item 9 score of 1 or greater.

#### ASQ Acute-Positive Escalation Procedures

Participants classified as acute positive on the ASQ were managed using a tiered emergency response protocol consistent with ASQ toolkit guidance. The responding clinician sought to keep the participant engaged by phone throughout the assessment and immediate safety-planning discussion. Concurrently, another member of the crisis intervention team communicated with the responding clinician through a dedicated instant messaging channel for imminent safety crises and contacted and engaged the emergency contact(s) the participant had provided at enrollment, as appropriate.

The crisis interventionist facilitated transport to the nearest emergency department or local psychiatric emergency service when an in-person evaluation was warranted and, when the participant could not be reached or their safety could not otherwise be confirmed, contacted local emergency resources to request a welfare check. The response was tailored to the participant’s clinical presentation and willingness to engage in safety planning, consistent with ASQ BSSA guidance [21].

### HITL Model Suicide-Risk Detection From Chat: Phase 2

#### Automated Conversational Safety Monitoring and Risk-Alert Routing

In phase 2, the screening and response protocol followed the same structure as in phase 1, with automated safety monitoring of participant interactions with Sona added. Sona was instructed to respond to direct disclosures of suicidal ideation or self-harm and to ask a direct safety question when indirect warning signs—such as hopelessness, perceived burdensomeness, veiled references to not being alive, giving possessions away, sudden calmness following depression, or social withdrawal—occurred in combination. After each interaction ended, the complete transcript was evaluated by a large language model–based routing classifier (Claude Opus 4.7; Anthropic). The current conversation transcript served as the primary evidence source; prior routing records, stored member memories, and up to 5 prior conversation summaries were supplied as contextual information but could not independently trigger an alert without supporting evidence in the current interaction. The classifier was instructed to generate a risk alert labeled CRISIS_988 when the current transcript contained evidence of suicidal ideation, current or imminent self-harm, or imminent harm to others. Model-generated alerts served as clinical triage triggers rather than automated determinations of suicide risk acuity; risk assessment and stratification were performed by a human clinician using the ASQ. Alerts were routed to the clinical safety Slack channel used for PHQ-9 alerts.

During business hours, the first available clinician responded to the alert by calling the participant and sending resources by email if the participant could not be reached. SMS text messaging was introduced in phase 2 as an additional outreach and ASQ administration modality to improve successful contact with participants who did not answer the phone. Outreach via SMS text message or email was considered successful if the participant responded. All participants who were reached and screened using the ASQ at baseline, as well as those with a new positive PHQ-9 item 9 screen at the 30-day follow-up, were contacted for ASQ screening 30 days after baseline.

To provide an external evaluation of the conversational suicidality detection pathway, the unchanged production classifier and version-controlled prompt were evaluated using the held-out test set of CRADLE-Dialogue [22], a benchmark comprising multiturn conversations independently annotated by 4 clinical experts. No benchmark conversations were used to modify the classifier. Dialogues classified by the clinical experts as containing ongoing active suicidal ideation comprised the target-positive group. The strict-negative comparison group comprised dialogues containing neither a confirmed ongoing crisis nor an alert-level signal. Sensitivity and specificity were calculated with 95% Wilson CIs.

#### Protocol Fidelity

A mandatory documentation and closed-loop confirmation system was used to support protocol fidelity. Clinicians were required to document all outreach attempts and their outcomes. They received repeated prompts until outreach completion was confirmed and documented, ensuring that alerts were not left unresolved and that adherence to the outreach sequence could be verified.

### Data Analysis

Participant characteristics, screening outcomes, and symptom severity were summarized using descriptive statistics. PHQ-9 and GAD-7 total scores were treated as continuous variables in descriptive analyses. Correlates of a positive suicidal ideation screen (PHQ-9 item 9 ≥1) were examined using logistic regression, with depressive symptom severity indexed by the PHQ-8 to avoid criterion contamination. Analyses were conducted first univariately and then using a multivariable model that included depression, anxiety, health-related quality of life, and age to adjust for confounding among these correlated measures. Demographic correlates were examined exploratorily, with no interaction terms tested and no correction for multiple comparisons. Demographic and 30-day follow-up data were analyzed using complete cases, without imputation, and the analytic n was reported for each estimate. Findings were robust to changes in the screen-positive threshold (item 9 ≥1 vs ≥2). Analyses were conducted using Python 3.11 (statsmodels; Python Foundation), with a 2-sided *P*<.05 considered statistically significant.

## Results

### Feasibility Outcomes

#### Screening Completion

Of the 784 individuals who downloaded the Sona app and provided digital consent in phase 1, 624 (79.6%) completed baseline suicide risk screening using the PHQ-9. In phase 2, 672 individuals downloaded Sona, of whom 650 (96.7%) completed baseline suicide risk screening using the PHQ-9. Tables 1 and 2 present the demographic and clinical characteristics, respectively, of the sample at baseline across phases 1 and 2.

**Table 1:** Demographic characteristics of the study sample^a^.

Variables		Phase 1 sample (n=624)	Phase 1 baseline PHQ-9 positive (n=81)	Phase 2 sample (n=650)	Phase 2 baseline PHQ-9 positive (n=133)
Demographic sample^b^, n		509	73	626	128
**Age (years)**					
	Mean (SD)	39.3 (10.3)	35.9 (9.8)	37.6 (10.0)	35.0 (8.5)
	Range	18-72	18-72	18-71	18-58
**Gender, n (%)**					
	Male	198 (38.9)	31 (42.5)	223 (35.6)	59 (46.1)
	Female	302 (59.3)	39 (53.4)	392 (62.6)	67 (52.3)
	Other	9 (1.8)	3 (4.1)	11 (1.8)	2 (1.6)
**Marital status, n (%)**					
	Married or partnered	245 (48.1)	16 (21.9)	255 (40.7)	49 (38.3)
	Single/never married	198 (38.9)	39 (53.4)	290 (46.3)	63 (49.2)
	Divorced	45 (8.8)	10 (13.7)	60 (9.6)	10 (7.8)
	Separated	12 (2.4)	3 (4.1)	12 (1.9)	4 (3.1)
	Widowed	5 (1.0)	1 (1.4)	9 (1.4)	2 (1.6)
	Missing/not available	4 (0.8)	4 (5.5)	0 (0.0)	0 (0.0)
**Income, n (%)**					
	Less than US $50,000	65 (12.8)	18 (24.7)	159 (25.4)	40 (31.3)
	US $50,000-US $99,999	218 (42.8)	39 (53.4)	275 (43.9)	56 (43.8)
	US $100,000-US $149,999	127 (25.0)	8 (11.0)	115 (18.4)	23 (18.0)
	US $150,000 or more	75 (14.7)	2 (2.7)	75 (12.0)	7 (5.5)
	Prefer not to answer/not classifiable	24 (4.7)	6 (8.2)	2 (0.3)	2 (1.6)
**Employment, n (%)**					
	Currently employed	393 (77.2)	50 (68.5)	600 (95.8)	118 (92.2)
	Not currently employed	113 (22.2)	21 (28.8)	26 (4.2)	10 (7.8)
	Missing/not available	3 (0.6)	2 (2.7)	0 (0.0)	0 (0.0)
**Education, n (%)**					
	High school	48 (9.4)	13 (17.8)	69 (11.0)	21 (16.4)
	Undergraduate	341 (67.0)	48 (65.8)	441 (70.4)	90 (70.3)
	Postgraduate	112 (22.0)	7 (9.6)	116 (18.5)	17 (13.3)
	Missing/not available	8 (1.6)	5 (6.8)	0 (0.0)	0 (0.0)
**Race/ethnicity, n (%)**					
	White/Caucasian	336 (66.0)	40 (54.8)	415 (66.3)	81 (63.3)
	Black/African American	51 (10.0)	5 (6.8)	74 (11.8)	13 (10.2)
	Hispanic/Latino	32 (6.3)	4 (5.5)	77 (12.3)	23 (18.0)
	Other or multiple races	90 (17.7)	24 (32.9)	55 (8.8)	11 (8.6)
	Missing/not classifiable	0 (0.0)	0 (0.0)	5 (0.8)	0 (0.0)

^a^Overall cohort sizes are shown in the column headings.

^b^“Demographic sample” reflects the total number of participants for each variable who completed the demographic survey. Percentages may not sum to 100% due to rounding.

**Table 2:** Clinical characteristics of the study sample^a,b^.

Variables	Phase 1 sample (n=624)	Phase 1 baseline PHQ-9 positive (n=81)	Phase 2 sample (n=650)	Phase 2 baseline PHQ-9 positive (n=133)
9-Item Patient Health Questionnaire, mean (SD)	6.8 (5.7)	15.7 (6.3)	10.1 (5.3)	15.7 (5.2)
7-Item Generalized Anxiety Disorder Scale, mean (SD)	6.1 (5.2)	12.7 (5.6)	9.2 (5.2)	12.9 (5.3)
Centers for Disease Control and Prevention 4-item Health-Related Quality of Life summary index (0-30), mean (SD)	13.5 (10.4)	22.9 (8.8)	19.9 (9.7)	24.5 (7.5)
Mentally unhealthy days (0-30), mean (SD)	9.9 (8.7)	18.4 (8.4)	15.5 (9.2)	20.9 (8.0)

^a^Clinical characteristics were summarized using all available observations for the corresponding instrument.

^b^Overall cohort sizes are shown in the column headings.

#### Alert Delivery

In phase 1, 81 of the 624 (13%) screened participants scored ≥1 on item 9 (the suicidality screening item) of the PHQ-9. All 81 positive screens generated a Slack notification to the clinical safety channel (100% delivery, with no reported alert delivery failures). In phase 2, 284 risk-alert events (234 PHQ-9–triggered events and 50 conversational events) generated notifications to the clinical safety channel, corresponding to 172 (26.5%) unique participants among the 650 screened participants. No documented instances of alert nondelivery were observed in either phase.

#### Time From Alert to Outreach Initiation

Among the 81 participants who screened positive on the PHQ-9 suicidal ideation item, human clinician outreach by telephone for further assessment was initiated after a mean of 11.2 minutes (SD 13.2 minutes; median 5.4 minutes; range 1-54 minutes). Of the 56 participants who did not answer the telephone and were subsequently contacted by email with crisis and mental health support resources, 2 (4%) responded with an acknowledgment of receipt.

#### Protocol Fidelity

Across the 2 study phases, all 365 alerts had fully documented outcomes and outreach attempt sequences in accordance with the clinical safety protocol, as verified by the closed-loop confirmation system described in the “Methods” section.

#### Unsuccessful Outreach

In phase 1, 25 of the 81 (31%) participants who screened positive on the PHQ-9 were successfully reached, whereas 56 (69%) were not. In phase 2, 73 of the 172 (42.4%) participants who triggered a safety alert were successfully reached, whereas the remaining 99 (57.6%) were not. Of the 98 successful outreach events in phase 2, 58 occurred by telephone, 28 by SMS text message, 5 by email, and 7 through an unspecified modality.

#### Staffing

Clinical team composition and the on-call structure are described in the “Methods” section. Staffing was adequate to address alert notifications in both phases. In phase 2, 11 of 284 (3.9%) alert events occurred during the weekend (after hours), including 3 of 234 (1.3%) PHQ-9–triggered alerts and 8 of 50 (16%) conversational suicidal ideation alerts. These alerts originated from 8 unique participants.

#### Technical Failures

No technical failures (ie, alert nondelivery or platform downtime) affecting the escalation pathway were formally logged during either study phase.

### Phase 1 Screening and Outreach Outcomes

Of the 81 participants who screened positive on the PHQ-9, 25 were successfully reached by telephone, and 22 agreed to further screening and completed the ASQ. Of these 22 participants, 7 answered “yes” to at least one of the first 4 ASQ screening questions, yielding a screen-positive rate of 32%. None of these 7 participants endorsed the fifth ASQ acuity item (“Are you having thoughts of killing yourself right now?”). Accordingly, all 7 cases were classified as nonacute positive. Of these participants, 2 (29%) denied current suicidal ideation but endorsed at least one previous suicide attempt. Among the 25 participants successfully reached, 18 (72%) reported receiving concurrent mental health treatment.

Of the 81 participants who screened positive on PHQ-9 item 9 at baseline, 47 (58%) completed the 30-day PHQ-9 follow-up. Of these, 25 (53%) had an item 9 score of ≥1, whereas 22 (47%) had a score of 0. Of the 7 participants who screened positive on the ASQ at baseline, 2 had no reported suicidal ideation at follow-up, scoring 0 on PHQ-9 item 9; 2 remained screen-positive, and 3 were lost to follow-up. Of the 2 participants who screened positive on the ASQ at baseline, remained positive on PHQ-9 item 9 at the 30-day follow-up, and were successfully reached for repeat ASQ administration, 1 screened negative on the ASQ, answering “no” to all 4 screening questions. The other screened nonacute positive but declined to complete the BSSA and safety plan. Of the participants who did not answer the telephone and were contacted digitally, 2 acknowledged receipt of the resources. Failure to establish clinical contact did not uniformly correspond to subsequent disengagement from the platform: 48 of the 56 (86%) participants who were never reached completed at least one conversation with Sona after their positive PHQ-9 assessment. A total of 27 (48%) participants remained active at least 7 days after the alert, and 17 (30%) remained active at least 30 days later. The 48 participants who remained active after the alert completed 444 subsequent conversations, with a median of 6 conversations per participant.

### Phase 2 Screening, Feasibility, and Outreach Outcomes

#### Risk-Alert Outcomes, Clinical Assessments, and Safety Interventions

At baseline, 133 of 650 (20.5%) participants screened positive for suicide risk on PHQ-9 item 9. During the 30-day postbaseline observation period, 172 of 650 (26.5%) participants triggered at least one risk alert. The chatbot workflow generated 284 risk-alert events among these 172 unique participants: 234 PHQ-9 item 9 events and 50 conversational suicidal ideation events. Successful outreach was documented for 98 alert events, involving 73 of the 172 (42.4%) participants (Figure 2). Time to the first clinician outreach attempt was calculable for 91 alerts (mean 11.5 minutes; SD 11.7 minutes; median 7 minutes; range 1-58 minutes). Among the 73 phase 2 participants successfully contacted following a risk alert, 44 (60%) reported already receiving mental health support, 18 (25%) reported no existing mental health support, and support status was unknown for 11 (15%).

**Figure 2:**
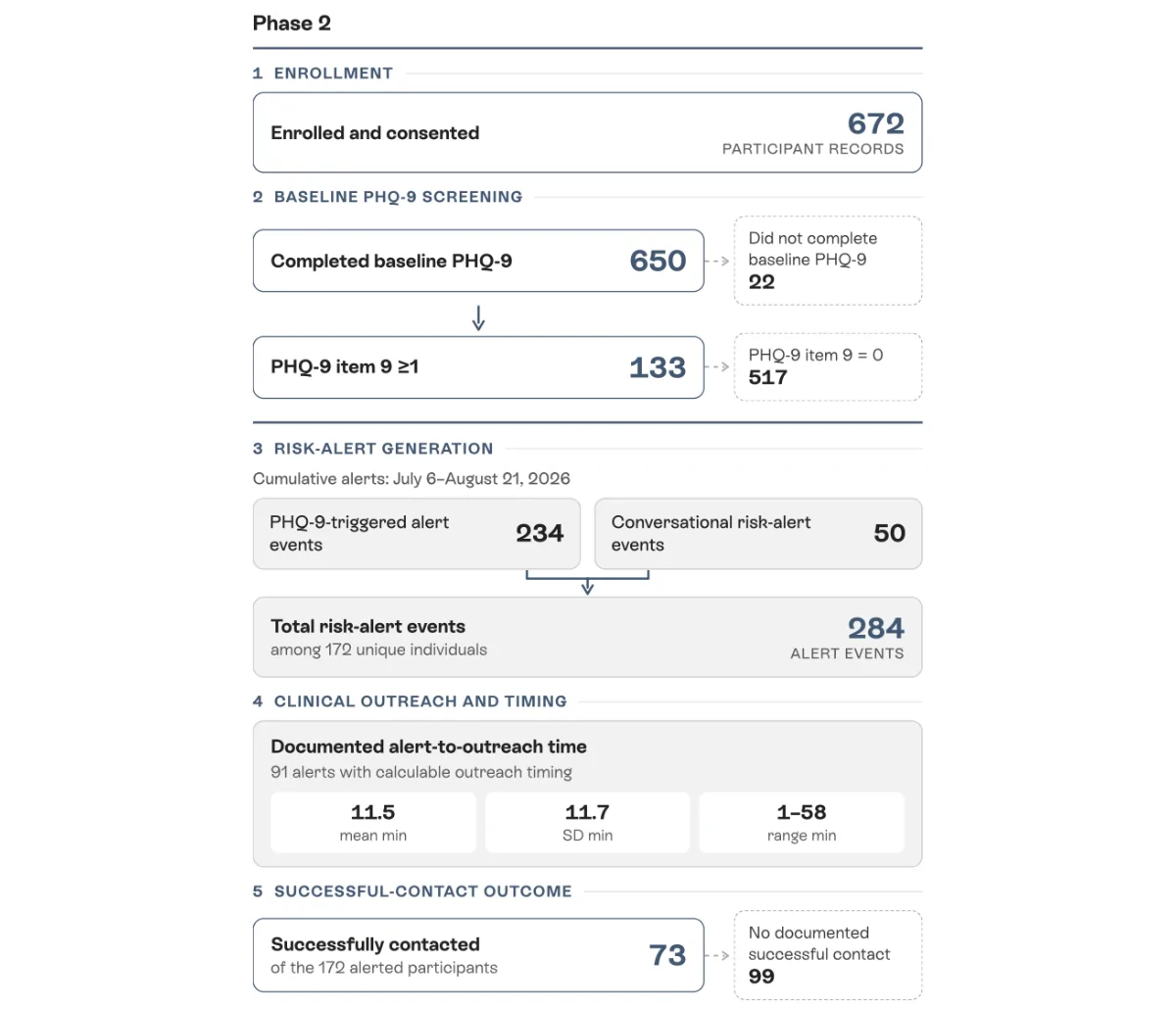
Baseline 9-item Patient Health Questionnaire (PHQ-9) screening, cumulative risk-alert events, and clinical outreach outcomes in phase 2. Risk alerts were triggered by a PHQ-9 item 9 score of ≥1 during the study window or by conversational risk detection. Thus, alerts were not restricted to participants who screened positive at baseline, and an individual could generate multiple alerts through either pathway.

Across baseline and the 30-day follow-up period, 71 complete numeric ASQ assessments were documented for 53 participants: 50 following positive PHQ-9 screens and 21 following conversational suicidal ideation alerts. Of these assessments, 30 (42%) were negative, 40 (56%) were nonacute positive, and 1 (1%) was acute positive (95% CI 0.04%-7.6%). The acute-positive assessment prompted the clinician to contact the local county crisis service, which arranged an in-person welfare check. In total, 24 BSSAs were conducted among 24 participants, and 16 safety plans were completed by 15 participants. One participant who screened positive on PHQ-9 item 9 at both baseline and the 30-day follow-up completed a safety plan after each screening, accounting for 2 of the 16 safety plans.

In phase 2, 55 of the 99 (56%) participants who were never reached completed at least one conversation with Sona after their first alert day; 41 (41%) participants remained active at least 7 days after their first alert. Among the 77 unreached participants with at least 30 days of observable follow-up, 19 (25%) remained active at least 30 days after their first alert. The 55 participants with postalert activity completed 546 subsequent conversations, with a median of 5 conversations per participant. Continued app use was an engagement measure only and did not establish participant safety, successful outreach, or the absence of unmet clinical needs.

Of the 133 participants who screened positive on PHQ-9 item 9 at baseline, 83 (62.4%) completed the 30-day PHQ-9 follow-up. Of these, 38 (46%) had an item 9 score of ≥1, whereas 45 (54%) had a score of 0. Of the 16 participants who screened positive on the ASQ at baseline, 7 (44%) had no reported suicidal ideation at follow-up, scoring 0 on PHQ-9 item 9; 5 remained positive on item 9, and 4 were lost to follow-up. Three participants who screened positive on the ASQ at baseline remained positive on PHQ-9 item 9 at the 30-day follow-up, and were successfully reached for repeat ASQ administration. Of these, 1 screened negative on the ASQ, answering “no” to all 4 screening questions, whereas the other 2 screened nonacute positive; both completed a safety plan.

#### Acute-Positive Case Summary

The participant with an acute-positive ASQ result had triggered a PHQ-9 alert at baseline. Multiple outreach attempts by telephone, email, and SMS text message were unsuccessful. At the 30-day follow-up, the participant again triggered a PHQ-9 alert and did not answer the telephone but responded to SMS text messages. During ASQ administration, the participant endorsed current suicidal thoughts and described having made concrete plans. The participant declined to complete a BSSA and safety plan. The crisis clinician provided crisis resources and unsuccessfully attempted to contact the participant’s designated emergency contact. Given the participant’s direct disclosure of current suicidal ideation and a concrete plan, the clinician judged the participant to be at potentially imminent risk and contacted the local county mental health crisis service to request a welfare check. The crisis service coordinated with local police, who made in-person contact with the participant. The participant denied suicidal intent, received information about local crisis services, and was not transported to an emergency department. The county crisis service subsequently conducted follow-up in accordance with its protocols.

#### Suicidality Detection Validation Analysis

In the external validation analysis, all 58 dialogues independently classified as containing ongoing active suicidal ideation generated a suicidal ideation safety alert, corresponding to a sensitivity of 100% (58/58; 95% CI 93.8%-100%). Among the 309 strict-negative comparison dialogues, 307 did not generate a suicidal ideation safety alert, corresponding to a specificity of 99.4% (307/309; 95% CI 97.7%-99.8%). Clinicians also reviewed the available conversations of study participants who endorsed PHQ-9 item 9 or triggered a conversational safety alert to identify suicidal ideation that had not been escalated. This targeted review identified no additional cases of ongoing active suicidal ideation that required an alert.

### HITL Suicide Screening and Assessment Acceptability

Qualitative interviews with participants who were contacted following a positive PHQ-9 screen revealed 2 distinct patterns: (1) participants appreciated being contacted by a human clinician, even when they did not feel they needed additional support; and (2) participants reported feeling surprised by the outreach. Notably, this surprise was reported even by participants who recalled seeing Sona’s disclosure that a human clinician might contact them. Several participants also indicated that they would have preferred a more explicit warning about the possibility of clinician outreach and suggested ways to implement such a disclosure. Qualitative feedback related to these findings is presented in Table 3.

**Table 3:** Interview participant quotes representing feedback related to clinician outreach.

Theme and participant ID		Quote
**Theme 1: Participants Appreciated Clinician Outreach**		
	Participant 3	“[The outreach] made me respect the app more because I knew that whatever information I was giving the AI was, in a way, being monitored...It made it even more real to me.”
	Participant 4	“At the very least, it is reassuring to know that there's a human on the other side of this...that was a very useful feature to differentiate this from all the other sort of therapy bots that are out there.”
	Participant 5	“I probably needed [the outreach] a little, not that I’d act on anything, but I felt like people were concerned about me, and that made me feel good.”
	Participant 7	“With so much misinformation out there, people want to know there’s an actual educated person they can be directed to if the app itself can’t handle something. I think a lot of people would specifically look for that.”
**Theme 2: Participants Were Surprised by Clinician Outreach**		
	Participant 1	“I didn’t think you guys would actually do it, just based on previous app experiences...I was kind of thinking I might get something, but it would be just an automated message, not a person-to-person. But it was nice that it was a person.”
	Participant 2	“I honestly didn’t really think it was a possibility. I thought it would have been like 100% ChatGPT, just, ‘Oh, if you’re feeling some way, call this number.’ I didn’t really think that there would be any physical backing behind the software.”
	Participant 6	“I wasn’t sure anyone would actually reach out, so I was a little surprised, especially since it was the same day...since I’ve never done a survey like this where someone actually reached out to me personally.”
	Participant 7	“It was really surprising because I wasn’t expecting it, especially not that quickly. Sometimes people promise to reach out and then it takes weeks, so having it happen within a 24-hour window was surprising and honestly a little heartwarming. Even though I personally wasn’t in crisis, I was just talking about my history, I think if it had been someone who was actually dealing with suicidal ideation in that moment, having a real person reach out would have meant a lot.”
**Suggestions for Implementing Additional Disclosure**		
	Participant 1	“[Implementing] at sign-up would be best. Not one of the thousand pages for [Terms of Service], but an actual page, like, ‘Hey, before you click this button, we might call you if we think it's necessary.’”
	Participant 4	“I wasn’t super comfortable handing out my [emergency contact] numbers to a person who called me out of the blue. So having some of that extra forewarning would have made me a little more comfortable doing that.”
	Participant 7	“Maybe some kind of expected wait-time indicator, so people have a sense of when they might be contacted...something like ‘you’ll be reached out to within this amount of time’ could help people feel a little less caught off guard.”

### Clinical and Demographic Correlates

In univariate logistic regression analyses, a positive suicidal ideation screen on PHQ-9 item 9 was significantly associated with greater baseline symptom severity across all clinical measures (*P*<.001 for all cases). Each additional point of depressive symptom severity, indexed by the PHQ-8 (the PHQ-9 excluding item 9 to avoid criterion contamination), was associated with 35% higher odds of a positive screen (odds ratio [OR] 1.35, 95% CI 1.27-1.42; *P*<.001). Anxiety severity (GAD-7; OR 1.29 per point, 95% CI 1.22-1.35; *P*<.001) and poorer health-related quality of life (CDC HRQOL-4; OR 1.71 per 5 additional unhealthy days, 95% CI 1.48-1.96; *P*<.001; n=555) were similarly associated with a positive screen. Screen-positive participants also reported roughly twice the symptom burden of screen-negative participants across these measures (see Table 2).

Among phase 1 participants with complete data for the demographic regression analyses, a positive screen was significantly associated with younger age (OR 0.66 per decade, 95% CI 0.51-0.88; *P*=.004) and lower educational attainment. Screen-positive rates showed a monotonic gradient across education levels (high school or less, 13/48, 27.1%; undergraduate, 48/341, 14.1%; postgraduate, 7/112, 6.3%; OR 0.43 per increasing education level, 95% CI 0.26-0.69; *P*=.001). Race/ethnicity was also associated with screening positive (non-White vs White: OR 1.72, 95% CI 1.03-2.89; *P*=.04). This association appeared to be driven by elevated odds among participants reporting multiracial, Asian, or other backgrounds. However, the combined and heterogeneous “other” category warrants cautious interpretation (OR 2.71 vs White, 95% CI 1.52-4.86; *P*=.001). Black (OR 0.68, 95% CI 0.23-1.99; *P*=.48) and Hispanic/Latino (OR 1.11, 95% CI 0.37-3.35; *P*=.85) participants did not differ significantly from White participants. Gender was not significantly associated with screening positive (men vs women: OR 1.23, 95% CI 0.73-2.08; *P*=.43), and current full-time employment showed a nonsignificant trend toward lower odds of a positive screen (OR 0.61, 95% CI 0.35-1.08; *P*=.09).

In a multivariable model including all 4 continuous measures, depressive symptom severity remained the strongest independent correlate of a positive screen (PHQ-8: OR 1.25, 95% CI 1.14-1.36; *P*<.001). Anxiety and health-related quality of life were no longer independently associated with a positive screen after adjustment, consistent with their high intercorrelations with depressive symptom severity (*r*=0.67-0.81). Younger age remained a borderline correlate (OR 0.97 per year, 95% CI 0.94-1.00; *P*=.05).

## Discussion

### Principal Findings

A HITL model of suicide risk screening, detection, and clinical escalation was feasibly implemented among adult users of a GenAI mental health chatbot. Although few studies are available for comparison, the positive screen rates observed in this study (81/624, 13%, in phase 1 and 133/650, 20.5%, in phase 2, among general and clinically symptomatic cohorts, respectively) were broadly consistent with rates reported in general outpatient mental health settings, which range from 9% to 15% [23-25]. In the clinically symptomatic cohort, the frequency of positive screens was higher than that reported in a previous study of a virtual behavioral health care setting using the ASQ (7.5%) [14], although the assessment instruments used to evaluate suicidality differed. Over the 30-day observation period, conversational disclosures and new PHQ-9 alerts increased the cumulative proportion of phase 2 participants who generated at least one risk alert (172/650, 26.5%). Taken together, these findings underscore the need for suicide risk screening and detection among individuals using this modality for psychological support. They also add to a growing body of literature suggesting that individuals may disclose sensitive mental health concerns more readily to AI-based or digital interventions because of reduced fear of judgment or stigma [8]. Across both phases, varying proportions of consenting participants completed suicide risk screening (ranging from 624/784, 79.6%, to 650/672, 96.7%), suggesting that universal screening may be feasible within the chatbot experience. After human clinician outreach modalities were expanded in phase 2 to include SMS text messaging in addition to telephone and email, 73 out of 172 (42.4%) alerted participants were successfully reached. This finding suggests that HITL escalation can reach a subset of individuals who may benefit from human-led crisis support, although more than half were not reached. Nevertheless, these findings should be considered in the context of the limited performance of existing tools: a systematic review of 29 mental health chatbots found that none met the criteria for acceptable detection and management of suicidal ideation [7], and general-purpose conversational AI tools in widespread use typically lack HITL response mechanisms. Thus, even incomplete contact may represent a meaningful improvement in clinical oversight compared with currently available consumer tools.

Across both phases, 1 of the 75 (1%) participants who completed the ASQ was classified as acute positive (95% CI 0.03%-7.2%). This case was appropriately escalated to local crisis intervention services, which arranged a welfare check. This outcome illustrates how the 2-tier ASQ risk stratification structure can identify acute risk warranting escalation beyond safety planning. However, the wide CI indicates that larger studies are needed to estimate the prevalence of acute risk in this population more precisely and to inform workforce capacity planning. This study also adds to the limited literature characterizing the mental health acuity of people who use GenAI mental health chatbots or consult general-purpose chatbots for psychological advice or support. Across 365 alert events, 1 prompted contact with emergency services, and none resulted in transport to an emergency department.

The majority of participants reached by a human clinician for further assessment of suicidal ideation reported that they were already receiving mental health treatment. This suggests not only that a sizable subset of chatbot users were managing a significant psychiatric burden but also that, in these higher-acuity cases, a GenAI chatbot served as an ancillary source of psychological support. Although these findings suggest that, with a standardized HITL protocol, individuals with clinically significant psychiatric symptoms can be monitored and contacted to address safety concerns while using a GenAI mental health chatbot, they do not establish whether such monitoring and outreach are clinically effective for this population.

Qualitative interviews with a subset of participants who were reached by a clinician provide preliminary support for the acceptability of this approach. Several participants described the outreach as reassuring and indicative of compassionate care, and some reported that clinician contact increased their trust in the Sona platform overall. However, several participants also reported being surprised by the outreach, including some who recalled seeing an initial disclosure that a clinician might contact them. These findings suggest that more explicit, proactive disclosure of the possibility of clinician outreach at enrollment may improve users’ preparedness for and comfort with this component of the safety protocol.

Although HITL models are widely recommended to promote safety among populations using or consulting GenAI tools for mental health support [9,10], published research to guide their implementation remains limited. This study provides preliminary evidence that universal suicide screening can be operationally implemented in a GenAI mental health chatbot serving both general and psychiatrically symptomatic populations and that a digital notification system can reliably generate alerts for a human crisis-response team, facilitating the initiation of outreach within a mean of 11-12 minutes after detection.

Given the documented risks of deploying GenAI tools without HITL oversight among high-acuity behavioral health populations [7,11-13], the 2-step pathway operationalized in this study offers a potential model for evaluation in other settings: (1) embedding a low-burden trigger (ie, PHQ-9 item 9), which is commonly administered for routine monitoring in standard practice [26]; and (2) conducting live clinical outreach by telephone for risk stratification (acute vs nonacute) and safety planning, as indicated, using the ASQ. Although varying proportions (25/81, 31%, in phase 1 to 73/172, 42%, in phase 2) of alerted participants responded to human clinical outreach via telephone or SMS text message, the response to email outreach providing crisis resources was minimal. These findings suggest that future studies and implementation models should prioritize telephone- and SMS text message–based outreach and explore other strategies for further safety assessment and intervention. Sustainably integrating this HITL escalation protocol into Sona’s standard operating infrastructure, rather than maintaining it as a study-specific procedure, will require dedicated clinical staffing capacity that scales with anticipated screening volume.

### Clinical and Demographic Factors

Demographic correlates of suicidality were generally consistent with prior literature [27,28], with positive screens observed more frequently among younger and less educated individuals. Greater depressive symptom severity was the most robust clinical correlate of a positive screen. Together with the higher rate of positive screens observed in this study than in other virtual behavioral health care and traditional outpatient mental health care settings [14,26], and the finding that most participants who screened positive reported receiving concurrent psychiatric care, these results suggest that individuals using an AI-based mental health chatbot may have greater psychiatric acuity than would be expected in a general population cohort. Accordingly, the risk profile identified in this study may help inform the development of clinical infrastructure to support HITL models of AI-delivered care.

### Limitations

Several limitations of this investigation warrant comment. First, participants resided in the United States, were English-speaking, and were employed and insured. Given that employment is a protective factor with respect to suicide risk [28,29], the positivity rate observed in this study may underestimate the prevalence of suicidality among adults using AI-delivered mental health support, among whom employment status may vary more widely. Likewise, these sample characteristics may be associated with greater access to care, as suggested by the high proportion of participants who reported current mental health care utilization (18/25, 72%, in phase 1 and 44/73, 60%, in phase 2), potentially reducing the need for emergency care services in the study cohort. Second, participants were recruited through online participant recruitment platforms, limiting the generalizability of the findings. Although rigorous screening and quality control practices were used to mitigate this limitation [30], this sampling approach can either suppress or inflate estimates of psychological symptom severity [31]. Replication of this study among individuals actively seeking mental health care or support is therefore warranted. Third, although the proportion of participants who screened positive for suicide risk and were successfully reached varied (ranging from 25/81, 30.9%, to 73/172, 42.4%), the lack of data on those who remained unresponsive to multiple outreach attempts by telephone and email limits our ability to fully characterize the positive-screen population. Future studies should explore alternative approaches to follow-up assessment among unreached participants, such as chatbot-delivered prompts or suicide safety screening tools, where appropriate.

In both phases, most participants with documented mental health care status were already receiving care (18/25, 72%, in phase 1 and 44/73, 60%, in phase 2). For these individuals, escalation pathways that involve their existing mental health provider may be preferable to emergency services when clinically appropriate and safe. Coordination with the treating provider may help maintain continuity of care, whereas routing individuals to emergency services may undermine trust, infringe on patient autonomy, and increase demands on emergency resources as screening programs expand. The sequencing of screening instruments also warrants consideration. In this protocol, the PHQ-9 was administered to all participants for 2 purposes: (1) to inform the Sona intervention approach by detecting depressive symptoms and (2) to serve as the initial suicide risk trigger and gateway to ASQ assessment. However, because PHQ-9 item 9 captures passive as well as active suicidal ideation, the threshold of ≥1 used in this study generated a substantial volume of alerts requiring human outreach, including many involving individuals already receiving mental health care. An alternative model would administer the ASQ digitally at intake, with human clinicians involved according to the screening outcome: immediate outreach for acute-positive screens and provision of safety planning resources and, where an existing mental health provider has been identified, coordination with that provider for nonacute-positive screens. The use of SMS text messaging for outreach and ASQ administration in phase 2 suggests that a digitally delivered, tiered approach may be feasible. However, self-administered digital delivery of the ASQ within a chatbot would require evaluation before implementation. Additionally, clinical diagnostic data were not collected, limiting our understanding of the clinical characteristics of both the positive-screen subgroup and the overall sample beyond depressive and anxiety symptoms and health-related quality of life. Substance use, trauma history, pain, and physical health status have been associated with suicide risk among populations seeking virtual care for substance use disorders [14]. Investigating whether these factors are also associated with suicide risk among individuals using GenAI for mental health support is therefore an important direction for future research. Moreover, although the conversational suicidality detection classifier was validated and showed promising sensitivity and specificity, the false-negative rate (ie, the proportion of potentially missed conversational disclosures) could not be quantified from these data, warranting replication and extension of this work. The study also used a brief, 30-day postbaseline observation period, leaving the longer-term effects of the crisis escalation procedures on mental health symptom resolution unknown. Finally, measurement reactivity may have affected the disclosure of suicidal ideation. Participants were informed before screening that endorsing safety-relevant symptoms could prompt clinician outreach, which may have led some individuals to underreport suicidal ideation. Similarly, an unknown proportion of participants who did not respond to clinician outreach may have been avoiding contact rather than being unavailable, potentially affecting both the study’s reach rate and 30-day follow-up participation. Graduated escalation designs that reserve direct clinician contact for higher-acuity disclosures may help reduce underreporting or avoidance of clinical contact.

### Conclusions

To our knowledge, this study was the first to implement and describe a HITL workflow for suicide risk screening, detection, and clinical escalation within a GenAI mental health platform. Suicide risk screening yielded positive screens in 81 out of 624 (13%) and 133 out of 650 (20.5%) participants in the general and clinically symptomatic adult cohorts, respectively. Importantly, the model described herein is a purpose-built mental health chatbot designed to incorporate and monitor clinically informed safety processes and guardrails. This study describes a safety protocol that may help consumers and clinicians assess the suitability of chatbots designed for psychological support compared with general-purpose conversational AI tools. Findings from this single-arm implementation study may inform the future development of HITL safety infrastructure for digital mental health, a rapidly evolving field in which evidence-based best practices remain limited.

## Data Availability

The data collected for this prospective implementation study are not publicly available because of the sensitive and confidential nature of mental health and suicide risk assessment data and the risk of participant reidentification. Deidentified data are available from the corresponding author (SG) upon reasonable request, subject to approval by the appropriate institutional review board and compliance with applicable restrictions on data disclosure.

## Appendix

Multimedia Appendix 1 Phone mockup plus screenshot of Sona, a GenAI mental health chatbot app.

Multimedia Appendix 2 Screenshot of Sona, a GenAI mental health chatbot app.

Multimedia Appendix 3 The iCHECK-DH (I Checked – Digital Health) checklist.

## Abbreviations

ASQ: Ask Suicide-Screening Questions
BSSA: Brief Suicide Safety Assessment
CDC HRQOL-4: 4-item Centers for Disease Control and Prevention Health-Related Quality of Life-4
GAD-7: 7-item Generalized Anxiety Disorder Scale
GenAI: generative AI
HITL: human in the loop
HL7 FHIR: Health Level Seven International Fast Healthcare Interoperability Resources
iCHECK-DH: I Checked – Digital Health
OR: odds ratio
PHQ-9: 9-item Patient Health Questionnaire
