# Supplementary material for: Human-in-the-Loop Suicide Risk Detection and Clinical Escalation in a Generative AI Mental Health Chatbot: Prospective Implementation Study
Source: JMIR Form Res. 2026 Oct 23;10:e106118. doi: 10.2196/106118 (PMC13743965; doi:10.2196/106118)
Supplement: Supplementary file 1 [file formative_v10i1e106118_app3.pdf]

iCHECK-DH Checklist

Human-in-the-Loop Suicide Risk Detection and Clinical Escalation in a Generative AI Mental Health Chatbot: Prospective Implementation Study

---

TITLE (Item 1 - Mandatory)

The title identifies this as an implementation study and describes the intervention.

✓ Reported in title.

ABSTRACT (Item 2 - Mandatory)

The abstract includes background, objective, methods, results, and conclusions, structured to reflect the two-phase design. Key elements reported: screening completion (Phase 1: 624/784 participants, 13.0% PHQ-9 item 9 positive; Phase 2: 650/672 participants, risk detection alerts among 26.5%), time to human response (Phase 1: mean 11.2 min, SD 13.2; Phase 2: mean 11.5 min, SD 11.7), and successful clinician contact (Phase 1: 30.9%; Phase 2: 42.4%).

✓ Reported in Abstract (p. 2-3).

INTRODUCTION

Item 3: Context (Mandatory)

The implementation took place in the United States, conducted fully remotely. Recruitment was conducted via Prolific and CloudResearch April 2026 (Phase 1) and July 2026 (Phase 2). The target population in both phases was English-speaking adults aged 18+ with smartphone access, currently employed or insured, residing in the U.S.; Phase 2 added a minimum symptom-severity eligibility criterion (PHQ-9 or GAD-7 >5). The implementation represents a two-phase pilot/evidence-generation program for Sona, a GenAI mental health chatbot. Social and organizational context includes the recognized gap between mental health treatment need and workforce capacity, and the proliferation of AI mental health tools without established safety standards.

✓ Reported in Introduction (p. 4) and Methods, Study Design and Participants (p. 6-7).

Item 4: Problem Statement (Mandatory)

The implementation addresses the absence of standardized, scalable procedures for detecting and responding to suicidal ideation in GenAI mental health chatbots. Despite best-practice recommendations for human-in-the-loop (HITL) oversight, most GenAI mental health tools lack such protocols, and existing chatbot evaluations show widespread failure to adequately detect and manage suicidal ideation.

✓ Reported in Introduction (p. 4-5).

Item 5: Similar Interventions (Mandatory)

This implementation builds on the authors' prior suicidality screening study in a virtual addiction clinic (Glasner et al., 2023; PMID: 37688670), conducted in collaboration with the NIMH. The added value of the current study is extension to a GenAI mental health chatbot population, use of an automated escalation system, a broader clinical and demographic characterization of the target population, and a conversational (chat-based) suicide risk detection pathway alongside the PHQ-9 screening pathway (Phase 2).

✓ Referenced in Methods, sample size justification (p. 7), and Discussion (p. 22-23).

METHODS

Item 6: Aim and Objectives (Mandatory)

Three pre-specified objectives: (i) characterize the frequency of suicidality, assessed by universal screening and conversational risk alerts, among adults receiving psychological support from a GenAI mental health chatbot; (ii) describe the feasibility (screening completion, alert delivery, time to outreach, contact success rates, and further clinical assessment) and acceptability (based upon qualitative data gathered from positive screen participants) of implementing a universal suicidality screening and a detection system with HITL clinical oversight; (iii) describe the emergency care needs of the target population based on suicidality risk stratification.

✓ Reported in Introduction (p. 5-6).

Item 7: Blueprint Summary (Mandatory)

The intervention is a two-phase, human-in-the-loop suicide risk screening and clinical escalation protocol embedded in the Sona GenAI mental health chatbot. In both phases, PHQ-9 item 9 serves as an automated trigger; any score >1 sends a real-time Slack notification to a licensed clinical team. In Phase 2, a second detection pathway was added: an LLM-based routing classifier (Claude Opus 4.7, Anthropic) evaluates the full conversation transcript after each chat session and generates a CRISIS_988 alert when it detects suicidal ideation, current or imminent self-harm, or imminent harm to others; these alerts route to the same clinical Slack channel. Clinicians administer the ASQ (by phone in Phase 1; by phone or text in Phase 2), conduct a BSSA if indicated, provide referrals, and follow up at 30 days. Phase 1 screening/coverage was business-hours-only; Phase 2 expanded clinical coverage to a 24 hour/5 day per week model. A structured escalation cascade (phone → email, with up to three follow-up phone attempts, plus text messaging as a second modality in Phase 2) is used for non-response.

✓ Reported in Methods, Suicidality Measurement, Risk Detection, and Escalation (p. 8-11), and HITL Model Suicide-Risk Detection from Chat: Phase 2 (p. 10-12).

Item 8: Technical Design (Mandatory)

The platform is Sona, a proprietary GenAI mental health chatbot designed to support psychological well-being and associated holistic wellness goals through personalized conversations delivered via voice or text. The PHQ-9-based suicidality risk detection system is embedded in the Sona platform and integrates with Slack for real-time clinical notification. In Phase 2, an LLM-based routing classifier (Claude Opus 4.7, Anthropic) was added to evaluate conversation transcripts for risk language and generate alerts.The unchanged production classifier and its version-controlled prompt were tested on the CRADLE-Dialogue held-out test set, with 95% Wilson CIs. Assessments were administered digitally within the app and via survey. Data were stored on secure, password-protected servers. The platform is a commercially developed, proprietary solution.

✓ Reported in Methods (p. 6; p. 8-11).

Item 9: Target (Mandatory)

Target population: English-speaking adults 18+, U.S. residents, currently employed or insured, with smartphone access, recruited via Prolific and CloudResearch. Phase 1 (April 2026): 784/1,388 who completed the pre-screen downloaded the app and consented, and 624 completed baseline measures. Phase 2 (July 2026): 672/1,000 who completed the pre-screen downloaded the app and consented, and 650 completed baseline measures; Phase 2 added an eligibility criterion of mild-or-higher symptom severity (PHQ-9 or GAD-7 >5). The implementation was fully remote/digital in both phases.

✓ Reported in Methods, Study Design and Participants (p. 6-7).

Item 10: Data (Mandatory)

Informed consent was obtained digitally prior to baseline assessment. No personally identifiable information is reported; data are presented in aggregate. Data were stored on secure, password-protected servers. The study received exempt status from the WCG IRB under 45 CFR 46.104(d)(3) (exemption granted December 14, 2025). Data are not publicly available due to the sensitive nature of suicide risk assessment data; de-identified data are available upon reasonable request, subject to IRB approval.

✓ Reported in Methods, Ethical Considerations (p. 7-8), and Data Availability (p. 27).

Item 11: Interoperability (Mandatory)

Covered in Methods. The Sona platform integrates with Slack via a real-time notification pathway that routes PHQ-9-triggered and, in Phase 2, conversational-classifier-triggered alerts to the clinical safety team channel. The manuscript specifies that this escalation pathway is a proprietary, closed-loop system rather than a standards-based interoperability framework, and does not currently use

interoperability standards such as HL7 FHIR for data exchange with external electronic health record systems.

✓ Reported in Methods, Suicidality Measurement, Risk Detection, and Escalation (p. 9).

Item 12: Participating Entities (Mandatory)

Implementing organization: Digital Therapeutics, Inc. (New York, NY), a digital therapeutics company. All authors are employed by Digital Therapeutics, Inc.; the corresponding author is additionally affiliated with the University of California, Los Angeles (Department of Psychiatry). The study was funded entirely by Digital Therapeutics, Inc, without government involvement or external partners. Intellectual property resides with Digital Therapeutics, Inc.

✓ Reported in author affiliations (p. 1) and Funding Statement / Conflicts of Interest (p. 27).

Item 13: Budget Planning (Mandatory)

The study was funded by Digital Therapeutics, Inc., with no external costs to participants.

✓ Reported in Funding Statement (p. 27).

Item 14: Sustainability (Mandatory)

As described in the Discussion, the manuscript notes that sustainably integrating this HITL escalation protocol into Sona's standard operating infrastructure, rather than maintaining it as a study-specific procedure, will require dedicated clinical staffing capacity that scales with anticipated screening volume, addressing next steps for operationalizing and scaling the protocol beyond the pilot phase.

✓ Reported in Discussion (p. 24).

IMPLEMENTATION / RESULTS

Item 15: Coverage (Mandatory)

The implementation was conducted nationally in the United States via a fully remote, app-based platform, across two recruitment cohorts. In Phase 1, 624 of 784 consented participants (79.6%) completed the pre-treatment suicide risk screening. In Phase 2, 650 of 672 consented participants (96.7%) completed the pre-treatment screening.

✓ Reported in Results, Feasibility Outcomes (p. 13).

Item 16: Outcomes (Mandatory)

Primary outcomes are reported separately for each phase. Phase 1: screening completion 624/784 (79.6%); PHQ-9 item 9 positive rate 81/624 (13.0%); phone contact rate 25/81 (30.9%); ASQ positive rate 7/22 (32%, among those who received the ASQ); all 7 non-acute positive; time to outreach mean 11.2 min (SD 13.2). Phase 2: screening completion 650/672 (96.7%); baseline PHQ-9 positive 133/650 (20.5%), rising to 172/650 (26.5%) cumulative over 30 days; 284 risk-alert events (234 PHQ-9-triggered, 50 conversational) among 172 unique participants (26.5%); contact rate 73/172 (42.4%); time to outreach mean 11.5 min (SD 11.7); of 71 ASQ assessments across baseline and 30-day follow-up, 30 were negative, 40 non-acute positive, and 1 acute positive, necessitating escalation to a local psychiatric emergency intervention team; 24 BSSAs and 16 safety plans were completed.

Commented [1]: update with manuscript

✓ Reported in Results (p. 13-19) and Table 1 (p. 14-15).

Item 17: Lessons Learned (Mandatory)

Findings suggested that automated real-time escalation and a dedicated notification channel supported feasibility of the HITL model. While 31% of those who screened positive on the PHQ-9 responded to live clinical outreach calls, the response to e-mail outreach with crisis resources was minimal. This suggests that future studies and implementation models should index on telephonic outreach and explore other potential strategies (such as the text messaging implemented as a second outreach modality in Phase 2) for further safety assessment and intervention.

Commented [2]: add text stat?

✓ Reported in Discussion (p. 24-25).

Item 18: Unintended Consequences (Non-Mandatory)

This study found that 72% (18/25) of Phase 1 participants reached by a human clinician for further assessment reported that they were already receiving concurrent mental health treatment. In Phase 2, 60.3% (44/73) were already receiving mental health support. This unanticipated finding suggests that, for a substantial subset of higher-acuity individuals, the GenAI chatbot functioned as an ancillary source of psychological support rather than a primary point of entry into care, with implications for platform targeting and resource allocation.

✓ Reported in Discussion (p. 23).

DISCUSSION

Item 19: Discussion (Mandatory)

The results from this study suggest that a HITL model of suicide risk screening, detection, and clinical escalation can be feasibly implemented in a population of adults receiving psychological support from a GenAI mental health chatbot. Positive suicidal ideation screen rates were consistent with those observed in general outpatient mental health clinic settings. Limitations of the present study include: generalizability (U.S.-only, English-speaking, employed-or-insured, Prolific/CloudResearch samples); the unknown acuity of the majority of alerted participants who were never reached; the need for a larger sample to characterize true emergency care needs, given that only one case across both phases was classified acute-positive; the absence of clinical diagnostic data; the use of PHQ-9 item 9 as the gate to the ASQ; the classifier's false-negative rate in the study can't be quantified; prevalence may be underestimated due to employment being protective; online recruitment may bias symptom reporting

Measurement reactivity and contact avoidance; and the lack of outcome data beyond the 30-day window.

✓ Reported in Discussion and Limitations (p. 22-26).

GENERAL

Item 20: General (Non-Mandatory)

IRB information reported (WCG IRB, exempt status under 45 CFR 46.104(d)(3), #20254520, exemption granted December 14, 2025). Conflicts of interest disclosed (all authors employed by and hold stock options in Digital Therapeutics, Inc.). Funding disclosed as a standalone Funding Statement, separate from Acknowledgments. A Generative AI Disclosure statement is included.

✓ Reported in Methods, Ethical Considerations (p. 7-8), and Generative AI Disclosure / Funding Statement / Conflicts of Interest (p. 26-27).
